# Chronic Exercise Accelerates Disease Progression in a Rat Model of Desminopathy

**DOI:** 10.1002/jcsm.70369

**Published:** 2026-11-05

**Authors:** Henning T. Langer, Natalie Haritonow, Aniket S. Joshi, Alec M. Avey, Alec Bizieff, Hussein Mohammad, Hermann Zbinden‐Foncea, Marc Hellerstein, Katrin Hahn, Werner Stenzel, Keith Baar, Agata A. Mossakowski

**Affiliations:** ^1^ Department of Geriatrics and Medical Gerontology Charité – Universitätsmedizin Berlin, Corporate Member of Freie Universität Berlin, and Humboldt‐Universität Zu Berlin Berlin Germany; ^2^ Department of Physiology and Membrane Biology University of California Davis California USA; ^3^ Neurobiology, Physiology and Behavior University of California Davis California USA; ^4^ Department of Nutritional Sciences and Toxicology University of California Berkeley USA; ^5^ Exercise Physiology and Metabolism Laboratory, School of Kinesiology, Faculty of Medicine Universidad Finis Terrae Santiago Chile; ^6^ Department of Neurology With Experimental Neurology Charité – Universitätsmedizin Berlin, Corporate Member of Freie Universität Berlin, Humboldt‐Universität zu Berlin, and Berlin Institute of Health at Charité – Universitätsmedizin Berlin Berlin Germany; ^7^ Department of Neuropathology Charité‐Universitätsmedizin Berlin, Corporate Member of Freie Universität Berlin, Humboldt‐Universität Berlin Berlin Germany

**Keywords:** chronic injury, desminopathy, intermediate filament, mechanical vulnerability, muscular dystrophy, protein aggregation myopathy

## Abstract

**Background:**

Desminopathy is a progressive myopathy and the most common intermediate filament disease in humans. Desmin is an essential part of the filamentous network that aligns myofibrils, anchors nuclei and mitochondria and connects the z discs to the sarcolemma. We challenged a rat model with a mutation in R349P *Des*, analogous to the most frequent R350P *DES* missense mutation in humans, through eccentric‐biassed exercise training.

**Methods:**

R349P Des rats (Des) and their wild type (WT) and heterozygous (HET) littermates were subjected to 4 weeks of downhill running. At the end of 4 weeks, muscles were collected and assessed histologically and biochemically.

**Results:**

Des rats demonstrated significantly lower running capacity compared to wild type and heterozygous littermates (*p* < 0.0001), which exacerbated over the course of the study (*p* < 0.001). Running performance in Des rats decreased by ~50% after 4 weeks of downhill running. Desmin‐positive skeletal muscle aggregates, a histopathological hallmark of desminopathy, were ~2‐fold higher after downhill running in the Des group (*p* < 0.05). On a protein level, we found a differential exercise response of Des compared to WT affecting various cellular pathways concerning autophagy, proteasomal activity, energy metabolism and cytoskeletal proteins.

**Conclusions:**

Our results suggest an impaired ability of Des muscle to adapt to chronic eccentric‐biassed exercise and a subsequent exacerbation of the functional and histopathological phenotype.

## 1 Introduction

Skeletal muscle fibres must withstand repeated cycles of contraction‐induced mechanical strain, a challenge that is met by an integrated network of cytoskeletal proteins and membrane repair mechanisms. The intermediate filament desmin plays a central role in linking myofibrils, mitochondria and the sarcolemma, thereby ensuring proper force transmission and stress distribution [1]. When desmin is altered, this scaffold is compromised, leading to myofibrillar disarray, impaired mitochondrial function and altered mechanical and regenerative properties [2, 3]. In parallel, the sarcolemma depends on specialized repair systems, including dysferlin‐ and annexin‐mediated vesicle recruitment, to reseal microlesions caused by eccentric contractions. Failure of these cytoskeletal and repair processes not only compromises structural integrity but also activates downstream stress pathways such as apoptosis, autophagy and the ubiquitin–proteasome system 4.

More than 60 disease‐causing variants in desmin that spread over the entire gene have been described [4]. The resulting desminopathies are clinically heterogeneous muscular dystrophies that are commonly associated with skeletal and cardiomyopathy [5, 6]. First symptoms of desminopathy (muscular weakness and wasting, cardiomyopathy with conduction anomalies and shortness of breath) occur between the second and fifth decade of life [6, 7]. Mutant desmin can form insoluble intracellular aggregates typical of all myofibrillar myopathies. Aggregates disrupt preexisting filamentous networks and have been described as ‘toxic’ [8, 9]. The aggregates also affect the protein quality control (PQC) system [10, 11]. When the PQC gets overwhelmed by aggregates, protein turnover is affected, and this can contribute to disease progression.

The most prevalent pathogenic variant in human desminopathy is caused by the exchange of arginine to proline at position 350 of the desmin gene (DES) (R350P) [12]. Desminopathies in humans are associated with impaired skeletal muscle integrity and susceptibility to mechanical stress [13]. Studying Des‐deficient rats provides insight into molecular and cellular mechanisms of muscle injury and repair, allowing evaluation of potential interventions. We previously engineered a CRISPR‐Cas9 knock‐in rat with the homologous mutation R349P. The rat model of desminopathy showed a relatively mild phenotype in young rats and lower susceptibility to sarcolemmal and cytoskeletal damage following acute eccentric exercise in older rats [14]. We hypothesized that the challenge of lacking functional desmin made them preadapted to mechanical stress and that a more chronic exercise stimulus was needed to trigger the pathophysiology. We hypothesized that rats subjected to a 6‐week treadmill‐running protocol that included 4 weeks of downhill running would accelerate the rate of disease progression. To test this hypothesis, we trained wild type (WT), heterozygous (HET) and homozygous (Des) mutant rats and determined the effect on running performance, histology and biochemistry.

Downhill treadmill running was chosen as a model to increase eccentric loading during locomotion and to induce contraction‐related mechanical strain in rodent hindlimb muscles. Repeated lengthening contractions during downhill running produce high mechanical strain on sarcomeres and their cytoskeletal anchorage. This paradigm is established for eliciting microdamage, fibre remodelling and activation of repair pathways in slow‐twitch, postural muscles that are otherwise relatively fatigue resistant [15]. To provide a standardized and reproducible approach to assess running capacity across experimental groups and exposition to the same relative progressive workload, we used the incremental load test (ILT) for training. By gradually increasing treadmill speed until exhaustion, the ILT yields objective performance metrics that allow direct comparison between healthy and myopathic animals.

To assess the effects of contraction‐induced damage and adaptation processes in desminopathic rats, we focused our investigation on the complementary mechanisms of cytoskeletal integrity, membrane repair and cellular stress responses.

## 2 Methods

### 2.1 Animals and Ethical Approval

All procedures were approved by the Institutional Animal Care and Use Committee of the University of California, Davis, which is an AAALAC‐accredited institution. Animal housing was in accordance with recommendations of the *Guide for the Care and Use of Laboratory Animals*. Male rats between 400 and 560 days of age were selected to study functional, histological and biochemical differences in the phenotype of CRISPR‐Cas9 created knock‐in animals carrying a homozygous variant in desmin (*Des*; *n* = 9), heterozygous (*HET*; *n* = 8) or healthy, wild type littermates (*WT*; *n* = 9). Animals were housed in 12:12 h light–dark cycles and fed ad libitum. For a detailed description of the generation and characterization of this desminopathy model, see Langer et al. [16].

### 2.2 Downhill Running

The experimental protocol of our study is summarized in the graphical abstract (Figure 1). The downhill running protocol was performed on a motorized treadmill (Exer‐3/6, Columbus Instruments, Columbus, OH). The speed of the treadmill was calibrated at the beginning of each week. The rear of the treadmill was equipped with a low‐voltage, electric stimulating grid with individual on/off switches per lane.

**FIGURE 1:**
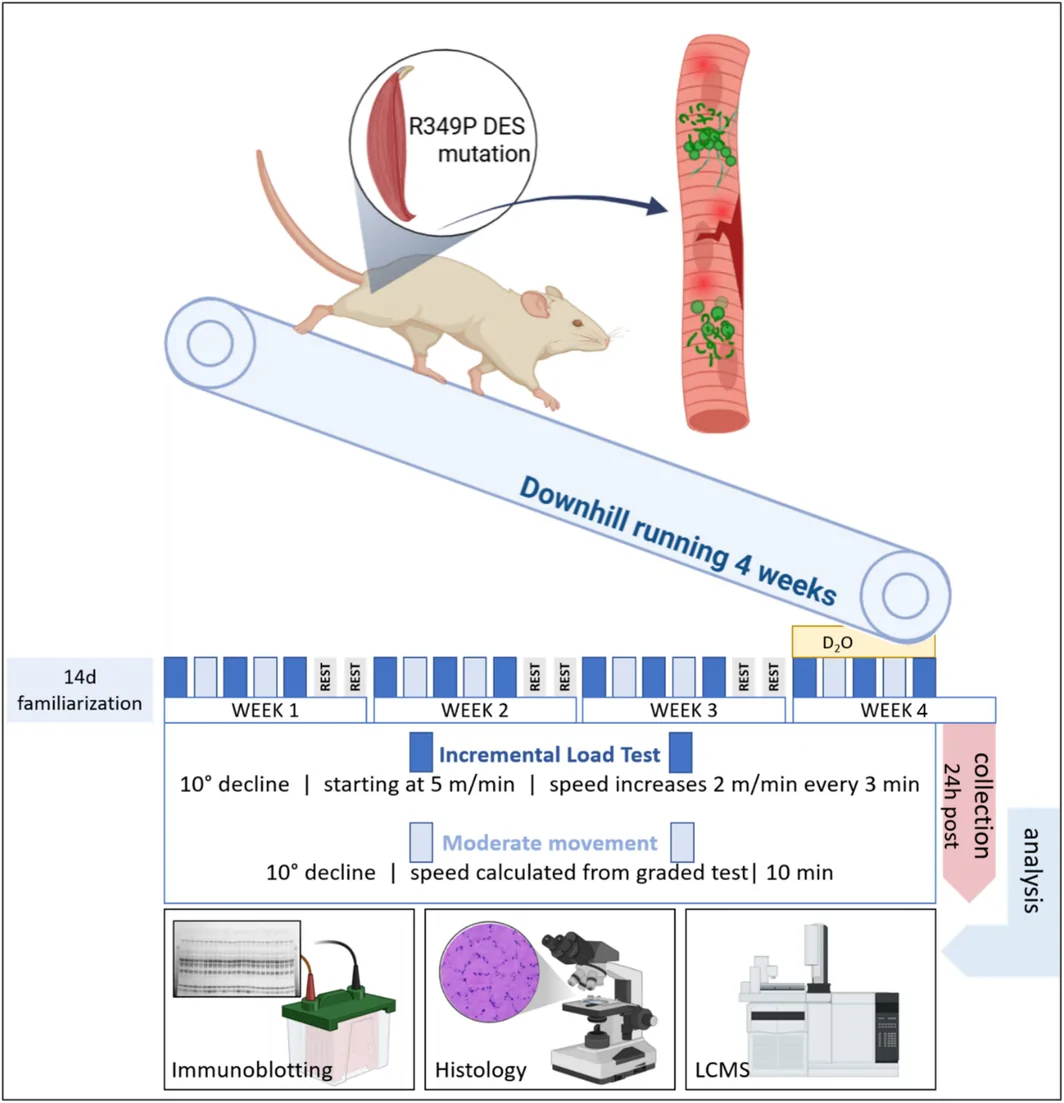
Study design and analyses of our 6‐week‐long rat study, with 2 weeks of acclimatization to the treadmill and 4 weeks of running intervention.

Rats were randomly assigned to sedentary (SED) or exercise (RUN) groups, and investigators were blinded to genotype. Prior to the exercise protocol, RUN rats (*n* = 14) underwent a treadmill familiarization (10 m/min at 0° decline for 10 min/day, 3 days/week for 2 weeks; 15 m/min at 0° decline for 10 min on Day 9). On Day 11, an incremental load test (ILT) was performed at 0° decline, starting at 5 m/min and increasing by 2 m/min every 3 min until exhaustion (protocol adapted from [17]). Following 3 days of rest, the 4‐week downhill running protocol was started.

During the downhill protocol, rats performed an ILT at −10° on Monday, Wednesday and Friday. To avoid overexercising but maintain a state of chronic physical activity, rats were walked at 40% of their respective maximal completed speed for 10 min on Tuesday and Thursday. Saturday and Sunday were rest days. Maximal speed and total running time were recorded for each ILT, whereas body weight was monitored weekly (Supporting Information S1: Figure S1).

All animals were euthanized 24 h after the final running session. One Des rat was sacrificed during Week 2 due to weight loss meeting humane endpoint criteria; postmortem examination revealed severe bowel obstruction.

### 2.3 Muscle Collection

The gastrocnemius (GAS), soleus (SOL) and tibialis anterior (TA) muscles removed under anaesthesia, blotted dry, weighed, transversally oriented under low magnification, pinned on cork at resting length and snap‐frozen in liquid nitrogen‐precooled isopentane at −120° in a steel container and stored at −80° in a freezer until further investigation. SOL muscle was used for all analyses.

### 2.4 Histology

Frozen SOL muscles were blocked, and serial cross‐sections (10 μm) were cut using a Leica CM 3050S cryostat (Leica Microsystems, Buffalo Grove, IL, United States). Haematoxylin and eosin (H&E) stains were performed as described previously [16].

For immunohistochemistry, muscle sections were incubated with a monoclonal desmin antibody (mouse, IgG2a, Santa Cruz Biotechnology, Dallas, Texas, 1:200 in NGS) or a polyclonal laminin antibody (rabbit, IgG [H + L], Sigma Aldrich, St Louis, Missouri, 1:500 in NGS) and the appropriate secondary antibodies.

Slides were imaged using a Leica DMi8 inverted microscope using the HC PL FLUOTAR 10×/0.32 PH1 objective (Leica Microsystems, Wetzlar, Germany) and using the LAS X software. For comparative analysis, exposure length remained fixed for all samples.

Muscle fibre properties and fibre types were analysed using FIJI and SMASH (MATLAB) software from the whole muscle sections.

### 2.5 Hydroxyproline Determination of Collagen Content

Collagen content was determined using a hydroxyproline assay [18].

### 2.6 Immunoblotting

SOL muscles were powdered on dry ice and prepared for western blots as described previously [16]. Briefly, total protein concentration of each sample was determined prior to electrophoresis using the DC protein assay (Bio‐Rad, United States), and equal amounts of protein were loaded per lane. Band intensities were normalized to total protein staining per lane as assessed via imaging of stain‐free Criterion TGX gels (Bio‐Rad, United States) or Ponceau S staining of the membrane. Densitometric analysis was performed using Image Lab (Bio‐Rad, United States), and values are presented as relative to the sedentary WT control group. The following antibodies were used in this study at a concentration of 1:1000: Cell Signaling (Cell Signaling Technology, Danvers, MA: microtubule‐associated proteins 1A/1B light chain 3B [LC3B] [#2775; lot 10], unc‐51 like autophagy activating kinase 1 [ULK1] [Ser757] [#14202; Lot 1]; p62 [#5114; lot 4]; Annexin A2 [#8235; Lot 2], caspase 3 [#9662], autophagy related 7 [Atg 7] [#8558]; ubiquitin [#3933, Lot 5], NOS [pan] [#2977]); Santa Cruz (Santa Cruz Biotechnology Inc., Dallas, TX): Dysferlin (#16635; lot H162), Desmin (#271677; Lot F1913), muscle LIM protein (mLIM)/cysteine and glycine‐rich protein 3 (mLIM) (#166930; lot E2814); septin 1 (#373925); and Abcam (Abcam Inc., Eugene, OR): total oxidative phosphorylation (OXPHOS) (MS604‐300).

### 2.7 Proteomics

Animals were stable isotope labelled with deuterium oxide starting 5 days prior to collection. Endogenous deuterium levels were raised to about 4% by a priming intraperitoneal injection of 0.014 mL * g^−1^ bodyweight deuterium oxide in 0.9% NaCl. Deuterium levels were maintained via drinking water (4% deuterium oxide). To determine how desminopathy affected protein turnover, fractional synthesis rates (FSR) of proteins were measured in gastrocnemius lysates via LC‐MS. No differences were observed between WT and heterozygous (HET) animals in the proteins investigated, so we restricted the protein analyses to WT and Des samples. A total of 42 proteins met analytical criteria for turnover kinetics and were common among all four groups. These were compared statistically. Detailed methods are described in Supporting Information S2. Results are shown in Supporting Information S2: Figures S1 and S2 and Table S1.

### 2.8 Statistics

Two‐way analysis of variance (ANOVA) or two‐way repeated measures ANOVA with a post hoc Tukey's multiple comparisons test was used to test the null hypothesis. Unpaired *t* test was used to show the difference in Desmin aggregates between sedentary and running Des animals. An alpha of *p* < 0.05 was deemed statistically significant, and a *p* value between 0.05 and 0.1 was called a trend. Data in the text are reported as mean ± standard deviation. Data in the figures are visually represented as scatter dot plot with error bars indicating standard deviation. All analysis was performed with GraphPad Prism Version 8 (La Jolla, CA, United States).

## 3 Results

### 3.1 Chronic Eccentric–Biassed Exercise Exacerbates Desminopathy

After 14 days of acclimatization, rats underwent an incremental load test (ILT). Initially, WT rats ran for a mean of 1032 ± 168 s, HET rats for 933 ± 244 s, and rats with a R349P *Des* mutation (Des) ran for 804 ± 188 s. We saw no correlation between body weight and running time (*R* ^2^ = 0.25; data not shown). The running performance between the Des and WT groups was similar at baseline and only started to become significantly different during the fourth ILT (day 8, *p* < 0.01). Following the 4 weeks of training, WT and HET animals maintained their running times, whereas running performance continued to decrease in Des rats. During the last ILT (Day 24), WT rats ran for a mean of 940 ± 206 s, HET rats for 876 ± 143 s and Des rats for 399 ± 122 s, with the difference between WT and Des being highly significant (*p* < 0.001). Overall, running performance in Des animals decreased by 50% over the course of 4 weeks (Figure 2).

**FIGURE 2:**
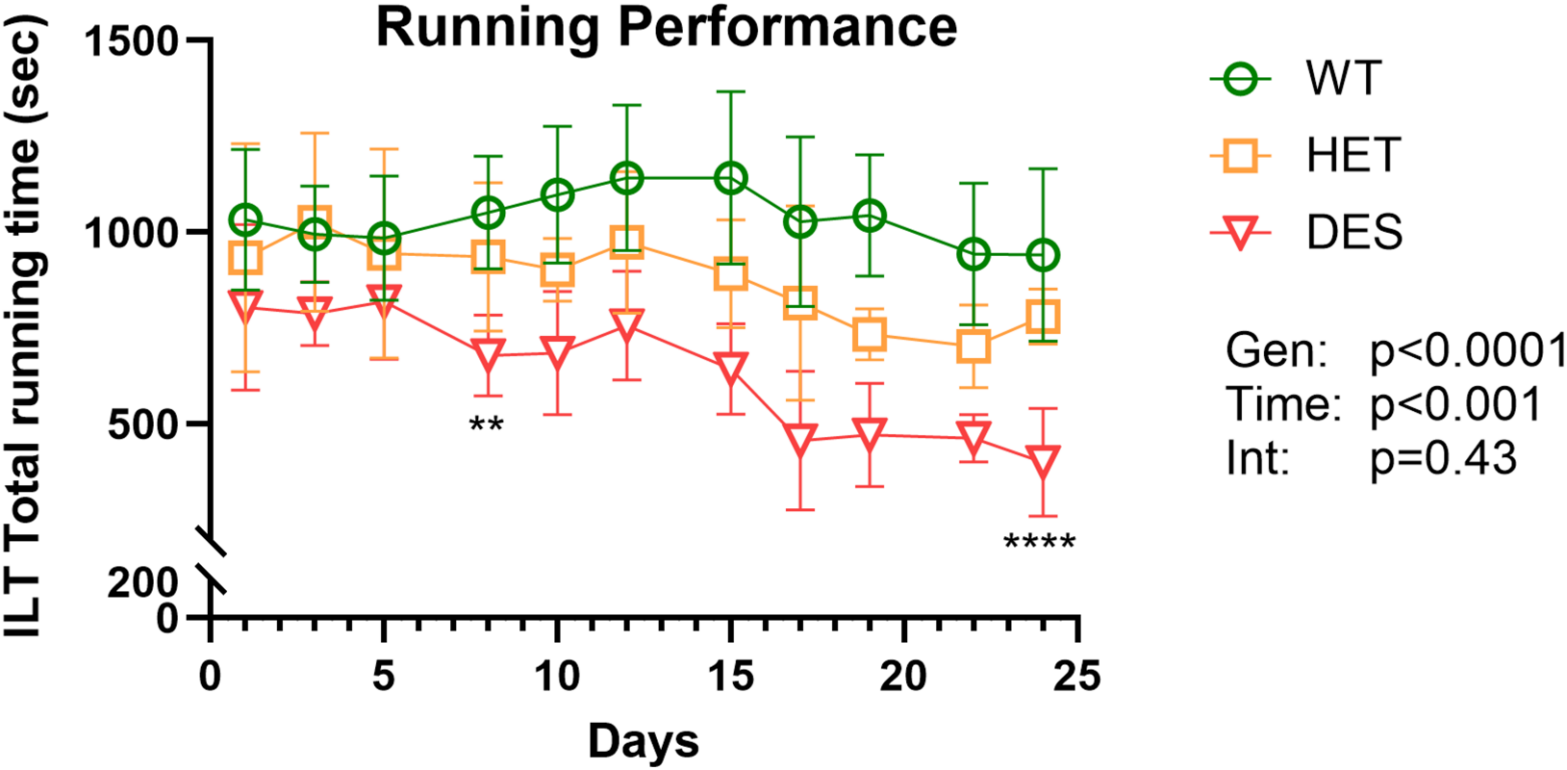
Incremental load test results over 4 weeks. Rats in the exercise groups were subjected to downhill running at a decline of −10° for 4 weeks after 2 weeks of treadmill familiarization. Rats performed an incremental load test (ILT) on Monday, Wednesday and Friday of each week. Treadmill speed was started at 5 m/min and increased by 2 m/min every 3 min until exhaustion. Graph shows the results for mean total running time (s) at each ILT. Gen, genotype; Int, interaction; Run, running; Time = effect of days of training; ** indicates *p* < 0.01, and **** indicates *p* < 0.0001 (two‐way repeated measures ANOVA, post hoc test of WT vs. Des). Error bars are SD.

### 3.2 Histopathological Signs of Myopathy in DES Animals

Histopathological changes consistent with myopathies, such as internalized nuclei, atrophic and necrotic fibres, and fibre size variability, were observed in H&E‐stained cross‐sections of sedentary Des soleus muscle (SOL) and slightly more pronounced in running Des SOL (Figure 3A). The percentage of fibres with internalized nuclei was significantly higher in Des compared to WT and HET at baseline (18% ± 5% of all fibres, 475 ± 73 absolute count, *p* < 0.0001 compared to WT and HET in post hoc analysis) and after running (15% ± 6% of all fibres, 315 ± 132 absolute count, *p* = 0.0002 compared to WT, *p* = 0.003 compared to HET) (Figure 3B). WT and HET fibres had a low amount of fibres with internalized nuclei, which did not change with running (WT SED 0.8% vs. WT RUN 1.4% ± 0.7%; HET SED 2% ± 0.7% vs. HET RUN 3% ± 0.7%).

**FIGURE 3:**
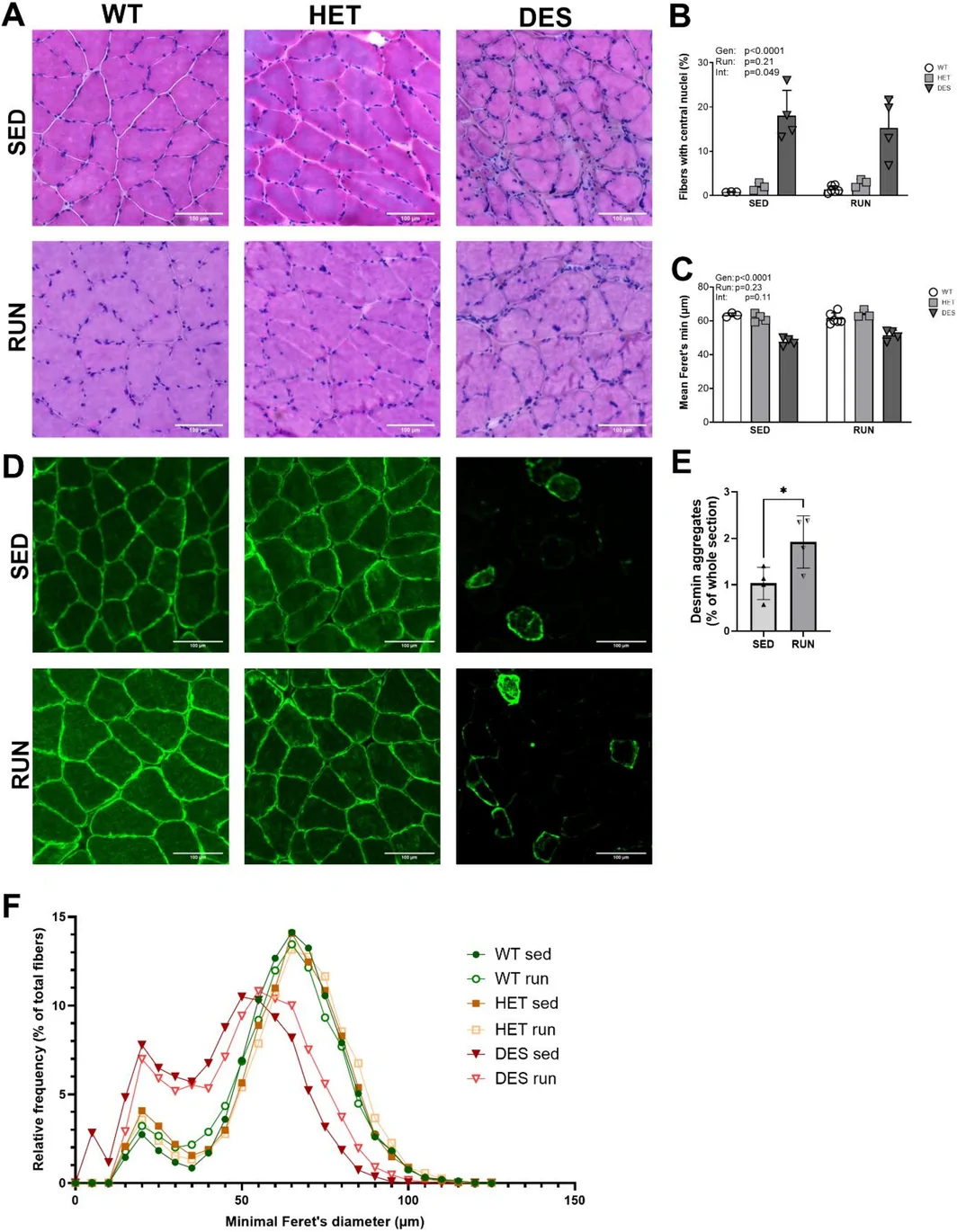
Histological changes in Des mutated muscle. H&E staining of soleus muscle (SOL) sections showed an increased number of necrotic and regenerating fibres (A) as well as an increased number of fibres with central nuclei (A&B) in desmin mutants. The mean minimal Feret's diameter calculated from all fibres of SOL cross sections was lower in Des compared to WT (C), and relative frequency analysis of all fibres showed a shift to a higher number of smaller fibres in Des (F). Immunohistochemical staining for desmin (green) showed distribution around the sarcolemma of WT and HET fibres but only aggregates in MUT animals (D). Desmin aggregates were quantified in relation to whole muscle section (E). Gen, genotype; Int, interaction; Run, running; AU, arbitrary units; Sed, sedentary controls. * indicates *p* ≤ 0.05.

Whole muscle cross sectional analysis (mean fibre count per cross section 2301 ± 415) showed significantly smaller fibres in sedentary DesES rats with a mean minimal Feret's diameter of 46 ± 1 μm compared to sedentary WT (63 ± 1 μm) and HET (53 ± 5 μm). Fibre size was not significantly impacted by running for any of the genotypes (Figure 3C). Frequency distribution analysis of Feret's diameter in whole muscle sections reveals a shift towards smaller fibres and a broader curve, suggesting increased size variability in Des (Figure 3F). Fibre size did not correlate with the change in performance between the first and last ILT (*R* ^2^ = 0.22, data not shown).

Immunohistochemical analysis of desmin showed subsarcolemmal and cytosolic desmin‐positive aggregates in Des animals (Figure 3D). Desmin aggregates relative to section area almost doubled in the running group (1.92% ± 0.49%) compared to the sedentary group (1.03% ± 0.3%, *p* = 0.04) (unpaired *t* test, Figure 3E).

The integrity of the sarcolemma was assessed by quantifying the number of fibres that stained positive for rat‐IgG. No myofibers of the sedentary animals showed signs of membrane damage, and only one DES animal had 0.13% IgG‐positive fibres in the running cohort, whereas all other sections were negative for IgG (data not shown).

### 3.3 Cytoskeleton and Membrane Repair

Sedentary HETs and Des animals showed reduced desmin protein levels (46% and 69% lower, respectively) compared to sedentary WT (Figure 4A). Running decreased desmin protein levels by 43% in WT rats. In contrast, desmin increased by 51% with running in HETs, with a similar trend being observed in Des rats (Figure 4A). However, the results in Des rats may have been affected by the overall low desmin protein levels bordering on the detection threshold. Dysferlin, a protein mainly associated with fusion of repair vesicles to the plasma membrane, tended to be higher in Des rats, but running had no effect on dysferlin levels (Figure 5A). Annexin A2, which interacts with dysferlin during membrane repair, did not differ between genotypes at baseline but increased with running (Figure 5B). Muscle LIM (mLIM), a protein prominent in the establishment and maintenance of the myofilament structure, was higher in sedentary HET (1.4‐fold) and Des (2.2‐fold) compared to WT (Figure 5C). Running increased mLIM in WT (2.1‐fold) and HET (1.7‐fold) but not in Des (Figure 5C). Septin 1 was elevated at baseline in sedentary Des rats. Running decreased Septin 1 only in Des, with no change in WT or HET (Figure 4C). Collagen content of whole muscle lysates did not differ significantly, although there was a trend towards a main effect of running (Figure 6E).

**FIGURE 4:**
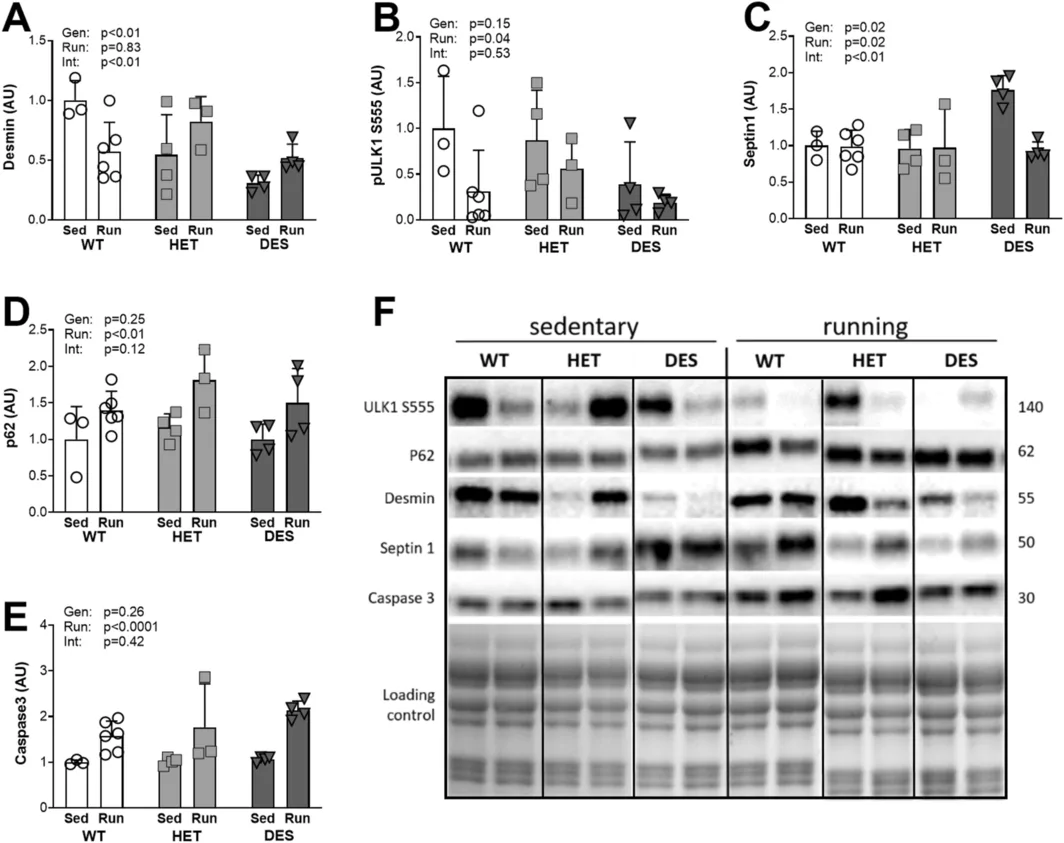
Western blots. Western blots of soleus muscle (SOL) of WT, HET and DES animals. (A) Desmin, (B) pULK555, (C) Septin1, (D) p62, (E) Caspase 3 and (F) representative blots. Loading control via Criterion TGX Stain‐Free Protein Gel image of total proteins. AU, arbitrary units; Sed, sedentary controls.

**FIGURE 5:**
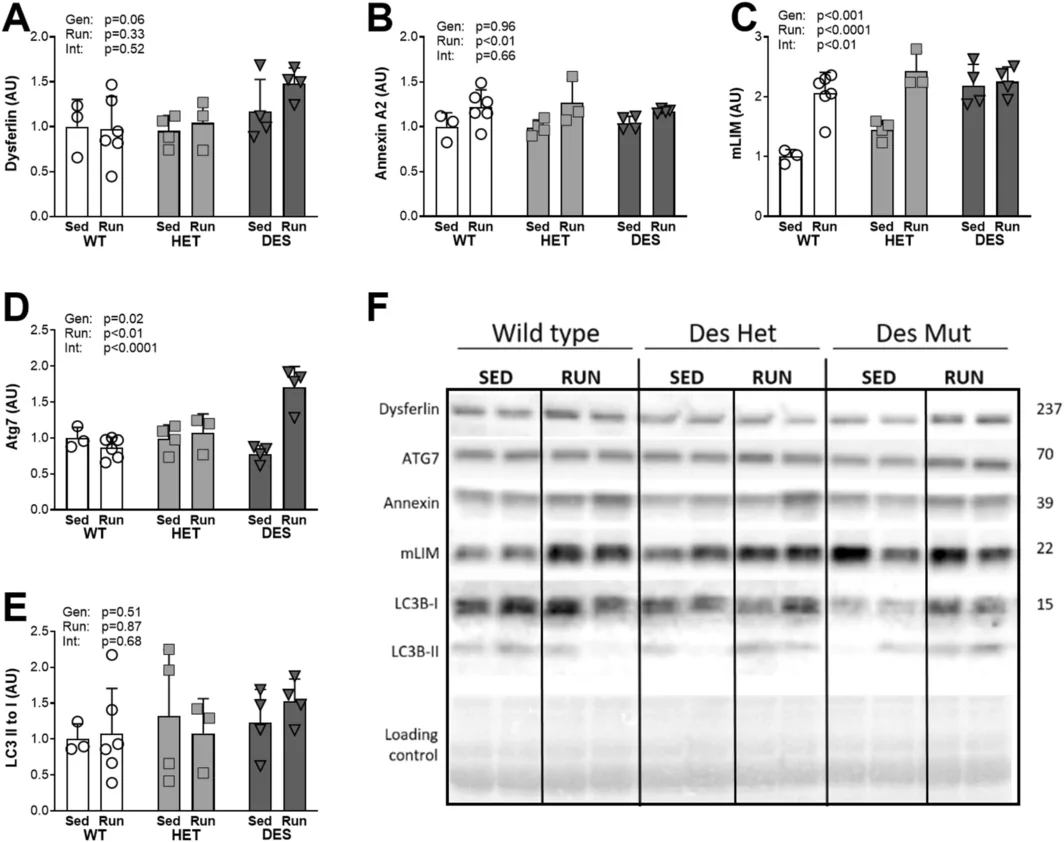
Western blots. Western Blots of soleus muscle (SOL) of WT, HET and Des animals. (A) Dysferlin, (B) Annexin A2, (C) mLIM, (D) Atg7, (E) LC3B II to I ratio and (F) representative blots. Loading control via Ponceau S membrane image of total proteins. AU, arbitrary units; Sed, sedentary controls.

**FIGURE 6:**
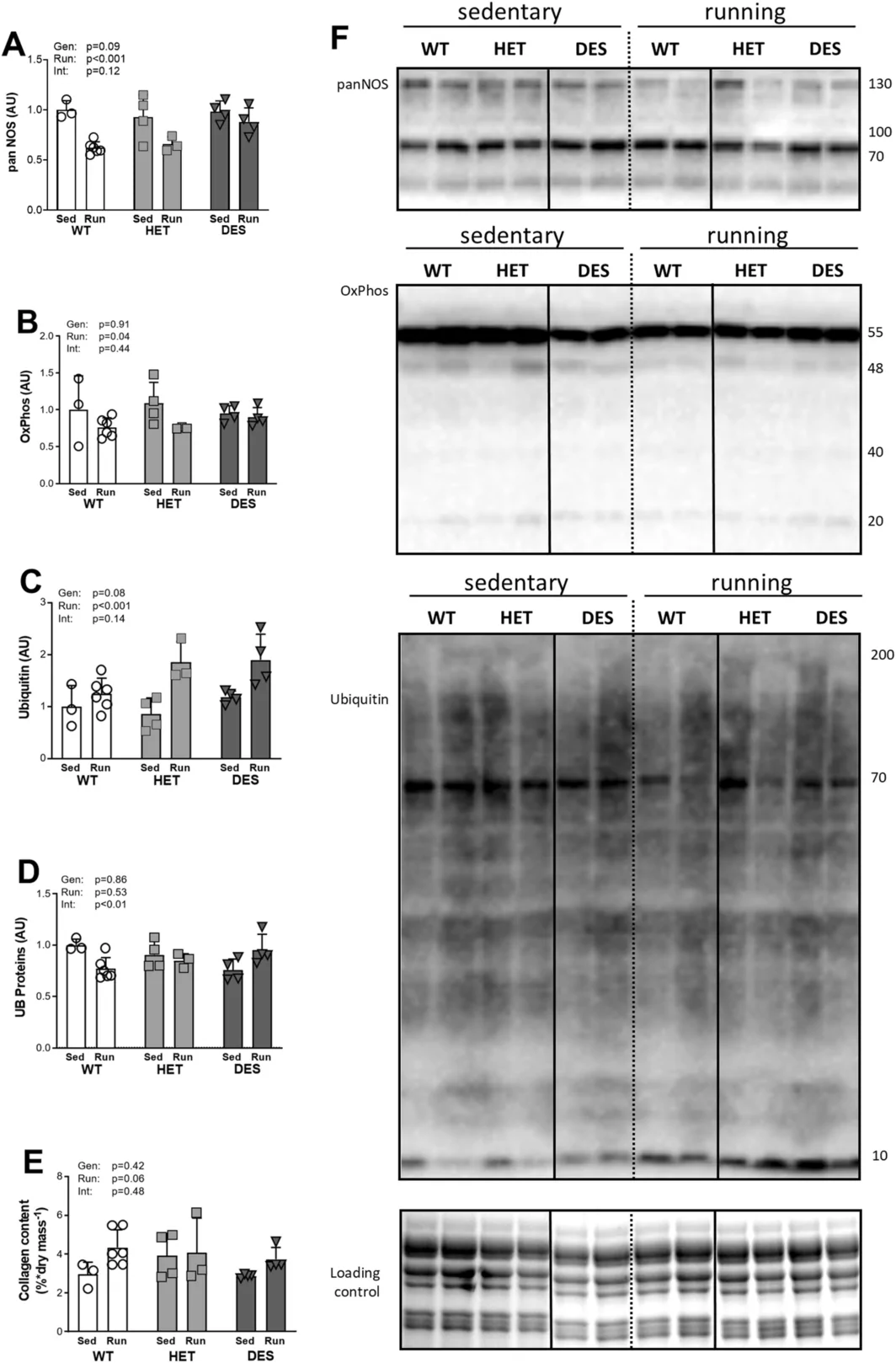
Western blots and collagen content. Western blots of soleus muscle (SOL) of WT, HET and Des animals. (A) Pan NOS, (B) OxPhos, (C) ubiquitin, (D) ubiquitinated proteins, (E) collagen content (determined via hydroxyproline assay) and (F) representative blots. Loading control via Criterion TGX Stain‐Free Protein Gel image of total proteins. AU, arbitrary units; Sed, sedentary controls.

Overall, baseline levels of cytoskeletal proteins were altered at the beginning of our intervention and with running in desminopathy, potentially reflecting increased baseline stress and stress‐adaptive mechanisms in desminopathic muscle.

### 3.4 Cell Damage, Degradation and Apoptosis

We next examined proteins involved in protein quality control and apoptosis. Upstream autophagy signalling through phosphorylated ULK1 (Ser555) tended to be lower in DES and decreased with running in all genotypes (Figure 4B). Atg7, associated with the ubiquitin‐proteasome system, slightly decreased in WT with running but increased 2.2‐fold in Des (Figure 5D). The LC3 II/I ratio, a marker of autophagy, did not differ between genotypes or with running (Figure 5E). p62, which binds ubiquitin and LC3, was similar at baseline across genotypes and increased in all groups with running (Figure 4D). Caspase 3, an apoptosis‐related protease, increased in running animals: 1.6‐fold in WT; 1.7‐fold in HET; and 2‐fold in Des (Figure 4E). Free ubiquitin increased with running in all groups, with a trend to rise more in groups with genetic alteration of desmin (Figure 6C). With running, ubiquitinated proteins declined by 23% in WT, remained stable in HET and rose by 26% in Des compared to sedentary controls (Figure 6D). NOS (pan) levels decreased in WT (~40%) and in HETs after running but remained unchanged in Des (Figure 6A). Total OxPhos showed a similar pattern (Figure 6B).

Overall, PQC and apoptosis markers responded differently depending on the genotype. Des rats showed stronger changes in Atg7 and ubiquitin dynamics, whereas caspase 3 increased in all genotypes, indicating that mutations in desmin affect the balance between proteostasis and apoptosis under mechanical stress.

## 4 Discussion

Here, we show that downhill running accelerates disease progression in Des rats, evidenced by reduced running capacity, impaired histological signs of tissue remodelling and a near‐doubling of desmin‐positive sarcoplasmic aggregates. Des animals ran only 40% as long as WT after training, underscoring that eccentric‐biassed exercise exacerbates, rather than ameliorates, muscle function.

The cellular and molecular mechanisms that lead from Des mutation to disease manifestation remain incompletely understood. There is conflicting data on the effects of mechanical stress on desminopathic muscle. Although most studies report increased mechanical fragility [13, 19], others suggest dysfunctional desmin could reduce susceptibility to membrane injury [20, 21, 22]. These discrepancies lead to speculation that other factors including mechanical stress drive disease progression [23]. The fact that serum creatine kinase (CK), a marker of muscle membrane damage, is elevated in < 60% of patients suggests that membrane injury is not the primary pathogenic mechanism [6]. Accordingly, we detected no membrane damage in sedentary or running Des animals, suggesting alternative mechanisms predominate.

We found only moderate histopathological alterations of skeletal muscle tissues after 24 days of running in Des animals. Sedentary Des rats showed a higher percentage of fibres with internalized nuclei compared to WT, consistent with previous findings [16]. After running, Des rats had fewer fibres with internalized nuclei, whereas fibre number per section and mean Feret's diameter remained unchanged, suggesting impaired adaptation to chronic exercise.

Most notably, desmin‐positive sarcoplasmic aggregates, the hallmark of desminopathy in humans, almost doubled after training in Des animals. Desmin aggregates are thought to promote disease progression by disrupting sarcomere architecture [8], impairing mitochondrial function [3] and affecting protein homeostasis and autophagy [24]. Aggregates escape degradation by the protein quality control (PQC) system, and over time, cellular debris becomes entangled, potentially ‘clogging’ autophagic and regenerative mechanisms. Desmin aggregates can also promote the aggregation of non‐aggregated desmin, thus expediting disease progression [9, 25]. To our knowledge, this is the first myofibrillar myopathy model that demonstrates accentuated formation of desmin‐positive protein aggregates.

Protein markers of muscle injury and repair mirrored these histological changes: Their baseline levels were higher in Des animals but did not increase further with running. Baseline mLIM was elevated in Des and increased in WT after running but not in Des. One important role of mLIM is to facilitate scaffolding at the Z‐disk, where it promotes the assembly of macromolecular complexes along sarcomeres and the actin‐based cytoskeleton [26]. An upregulation of this Z‐band associated protein after downhill running would be in line with previous findings that eccentric‐biassed exercise leads to adaptation at the Z‐band [27]. Protein aggregates in desminopathy contain debris of other proteins, including dysferlin [28], matching our observed trend of increased dysferlin in Des rats after running. Additionally, we saw a marked increase in Septin1 in sedentary Des rats that decreased after running. This finding may point towards a preadaptive response to mechanical stress that is exhausted by eccentric‐biassed exercise. The role of septins in desminopathy poses an intriguing research objective for future experiments, because they have been linked to other protein aggregation diseases like Parkinson's and Alzheimer's disease.

To further investigate the regulation of protein aggregation in our rats, we measured proteins involved in protein quality control (PQC). ULK1 initiates autophagy and is positively regulated through phosphorylation at Ser^555^ [29]. Single bouts of exercise have been shown to increase ULK1 phosphorylation at Ser^555^ both in mice and humans [30], but phosphorylation did not change after resistance exercise training in rats [31]. We saw lower phosphorylation of ULK1 at Ser^555^ in Des animals compared to WT, and there was a main effect for decreased phosphorylation with running. Downstream from ULK1, we also did not identify stark changes in PQC‐related proteins in Des animals. The exceptions were Atg7, which doubled in Des while decreasing in WT, and the total amount of ubiquitinated proteins, which decreased in WT but increased in Des. Together, these data could indicate that protein degradation may either be impaired or simply does not occur at the necessary rate to avoid protein aggregation in Des mutant muscles. However, follow‐up experiments are required to investigate this question more directly and answer which PQC mechanisms are primarily responsible for degradation of desmin aggregates.

Muscle remodelling after exercise and injury is known to be controlled by muscle protein turnover [32]. Stable isotope labelling revealed modestly elevated baseline protein turnover in gastrocnemius tissue of DES rats, which did not increase after training, matching histology and western blot data (Supporting Information S2: Figures S1 and S2 and Table S1). This is in line with our previous observations after acute eccentric exercise [14]. Taken together, this suggests that muscle remodelling is elevated at baseline in Des rats and does not adapt in response to external stimuli.

An important limitation of our study is that human desminopathy is typically autosomal‐dominant, whereas autosomal‐recessive cases are very rare. These rare cases are characterized by a near‐complete absence of desmin expression in striated muscle cells and appear to involve a different disease mechanism without surplus protein aggregates [33]. In our previous work, we focused only on homozygous and wild‐type animals due to the mild phenotype observed in HET animals. In this study, we hypothesized that eccentric‐biassed exercise might induce a more severe phenotype in HET animals. Although we observed molecular differences and differences in running performance, there were no significant histological differences between WT and HET groups. In a well‐established mouse model of the same mutation, homozygous (HOM) R349P knock‐in mice show markedly reduced desmin levels compared to WT and HET animals. In HOM R349P mice, there is a complete absence of wild‐type desmin and only low levels of mutant R349P desmin, which is incapable of forming a functional desmin network, leading to the formation of desmin‐positive protein aggregates [8]. Further studies are necessary to comprehensively compare the desmin network composition and aggregation mechanics in our rat model with those in the mouse model and human desminopathy cases to improve our understanding of the translational relevance of our findings. Additionally, treadmill running is an integrated performance measure that is comprised of more than soleus or gastrocnemius function. Other skeletal muscle groups such as the quadriceps are heavily involved as well and analyses of such muscles on the histological and biochemical level might reveal a divergent response to eccentric‐biassed exercise. Finally, any endurance performance is heavily reliant on cardiac muscle morphology and performance. Because desmin is strongly expressed in the heart and known to affect cardiac function [2], it is possible that the robust decline in running performance in our rats is not just a function of peripheral muscle decline but also progressive cardiomyopathy. Future projects need to more directly investigate the role of the heart in our desmin model.

In summary, downhill running reduces running capacity and exacerbates desmin aggregation in desminopathic rats. These findings highlight the importance of tailoring exercise recommendations to desminopathy patients, given the potential for harm and because physical therapy is currently the only prescribed therapeutic approach for patients with this disease. Until we have better progress and outcome measures for monitoring human desminopathy, we find that the prompt aggravation of disease with downhill running in our R349P Des rat makes it a convenient model for testing of new therapies. Future studies will have to determine whether therapeutic approaches targeting protein aggregates and PQC can lead to improved muscle function and ameliorate disease progression in desminopathic rats.

## Funding

This study was supported by the National Institute of Aging (R01AG056999 and R01AG45375) and Deutsche Forschungsgemeinschaft.

## Conflicts of Interest

The authors declare no conflicts of interest.

## Supporting information


**Data S1:** Supporting Information.

**Data S2:** Supporting Information.
