# Supplementary material for: Chronic Exercise Accelerates Disease Progression in a Rat Model of Desminopathy
Source: J Cachexia Sarcopenia Muscle. 2026 Nov 5;17(6):e70369. doi: 10.1002/jcsm.70369 (PMC13740671; doi:10.1002/jcsm.70369)

Endurance Exercise Accelerates Disease Progression in Desminopathy.

Henning T. Langer[2,3], Alec M. Avey[3], Alec Bizieff[5], Hussein Mohammad[5], Hermann Zbinden-Foncea[6], Marc Hellerstein[5], Keith Baar[3,4], and Agata A. Mossakowski[1,3]

[1]Department of Neurology with Experimental Neurology, Charité – Universitätsmedizin Berlin, corporate member of Freie Universität Berlin, Humboldt-Universität zu Berlin, and Berlin Institute of Health at Charité – Universitätsmedizin Berlin

[2]Department of Geriatrics and Medical Gerontology, Charité – Universitätsmedizin Berlin, corporate member of Freie Universität Berlin and Humboldt-Universität Zu Berlin, Berlin, Germany

[3]Neurobiology, Physiology and Behavior, University of California, Davis, CA

[4]Department of Physiology and Membrane Biology, University of California, Davis, CA

[5]Department of Nutritional Sciences and Toxicology, University of California, Berkeley, USA.

[6]Exercise Physiology and Metabolism Laboratory, School of Kinesiology, Faculty of Medicine, Universidad Finis Terrae, Santiago. Chile.

Corresponding author:
agata-anna.mossakowski@charite.de

Supplementary Materials 1

Body and muscle mass

DES rats weighed less than their WT and HET littermates at an average of 564±63 g (DES) compared to 637±32 g (WT) and 657±71 g (HET) (FigS1A) (p=0,02 for genotype, p=0.31 for running and p=0.92 for interaction).

Muscle mass of soleus (SOL), gastrocnemius (GAS), extensor digitorum longus (EDL) and tibialis anterior (TA) muscle was lower in DES rats (not shown), but not when calculated relative to body weight (FigS1B-E). Contrary to our expectation, muscle weights relative to body weight tended to decrease following 6 weeks of running in GAS (Fig1C, p=0.092 for genotype, p=0.03 for running and p=0.84 for interaction) and EDL (Fig1D, p=0.8 for genotype, p=0.05 for running and p=0.87 for interaction), and TA (Fig1E, p=0.29 for genotype, p=0.06 for running and p=0.61 for interaction). SOL muscle mass tended to increase in WT but decrease in DES, but again without reaching statistical significance (FigS1B, p=0.85 for genotype, p=0.59 for running and p=0.12 for interaction).

Figure S1 – Body weight and muscle mass. DES rats had lower body weight than WT and HETs (A). Muscles were collected 24h after the last run. Weights are presented relative to body weight (bw). SOL = soleus, EDL = extensor digitorum longus, GAS = gastrocnemius, TA = tibialis anterior muscle (B-E). Gen=Genotype, Run=Running, Int=Interaction. SED=sedentary controls, RUN=running animals.

Supplementary Figure 1

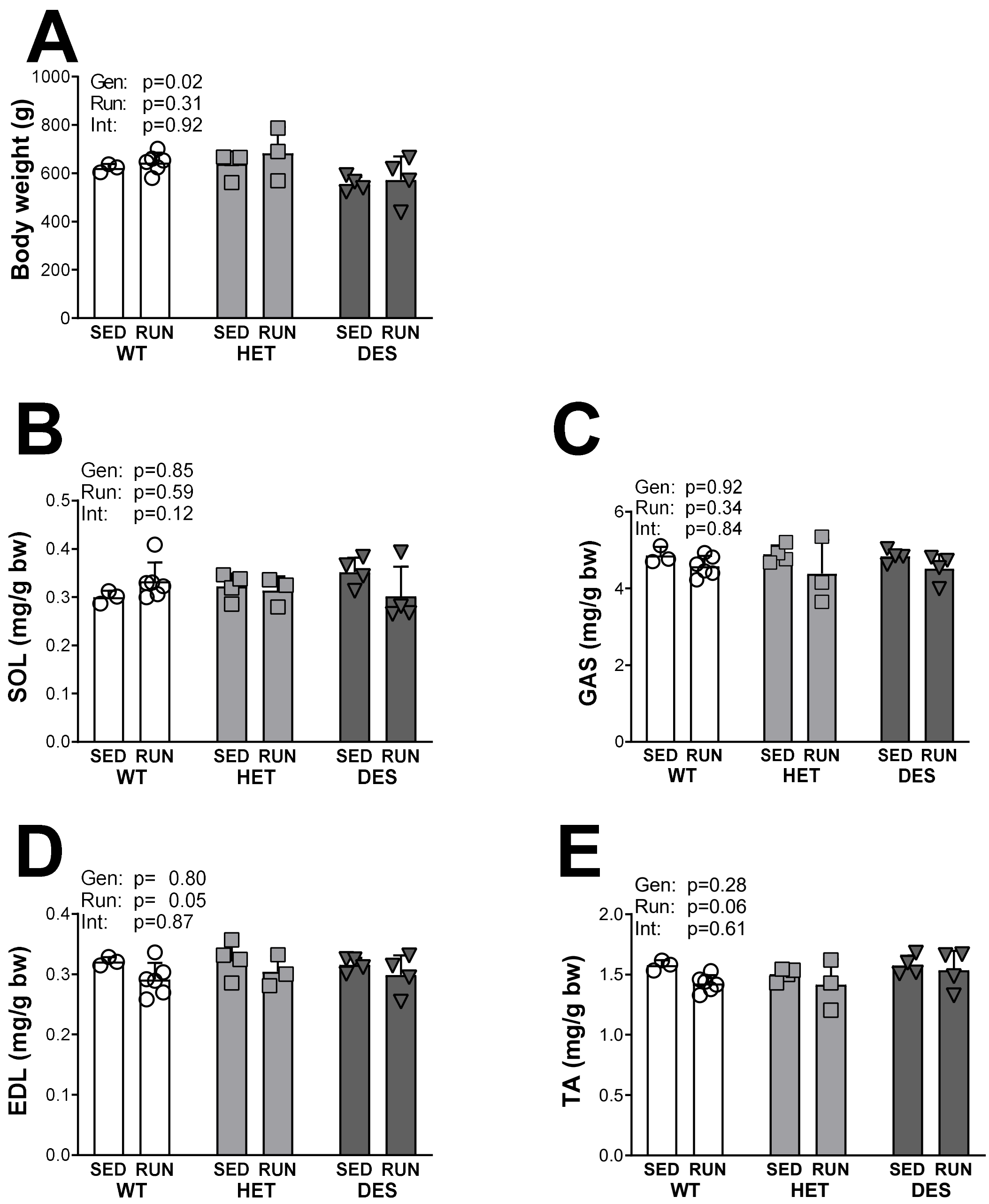

Supplement: Supplementary file 1 [file JCSM-17-e70369-s001.pdf]
