# Supplementary material for: Chronic Exercise Accelerates Disease Progression in a Rat Model of Desminopathy
Source: J Cachexia Sarcopenia Muscle. 2026 Nov 5;17(6):e70369. doi: 10.1002/jcsm.70369 (PMC13740671; doi:10.1002/jcsm.70369)
Supplement: Supplementary file 2 [file JCSM-17-e70369-s002.pdf]

Endurance Exercise Accelerates Disease Progression in Desminopathy.

Henning T. Langer[2,3], Alec M. Avey[3], Alec Bizieff[5], Hussein Mohammad[5], Hermann Zbinden-Foncea[6], Marc Hellerstein[5], Keith Baar[3,4], and Agata A. Mossakowski[1,3]

[1]Department of Neurology with Experimental Neurology, Charité – Universitätsmedizin Berlin, corporate member of Freie Universität Berlin, Humboldt-Universität zu Berlin, and Berlin Institute of Health at Charité – Universitätsmedizin Berlin

[2]Department of Geriatrics and Medical Gerontology, Charité – Universitätsmedizin Berlin, corporate member of Freie Universität Berlin and Humboldt-Universität Zu Berlin, Berlin, Germany

[3]Neurobiology, Physiology and Behavior, University of California, Davis, CA

[4]Department of Physiology and Membrane Biology, University of California, Davis, CA

[5]Department of Nutritional Sciences and Toxicology, University of California, Berkeley, USA.

[6]Exercise Physiology and Metabolism Laboratory, School of Kinesiology, Faculty of Medicine, Universidad Finis Terrae, Santiago. Chile.

Corresponding author:
agata-anna.mossakowski@charite.de

Supplementary material 2

Protein flux in skeletal muscle

To determine how desminopathy affected protein turnover, fractional synthesis rates (FSR) of proteins were measured via LC-MS. No differences were observed between WT and heterozygous (HET) animals in the proteins investigated, so we restricted the protein analyses to WT and DES samples. A total of 42 proteins met analytical criteria for turnover kinetics that and were common among all 4 groups. These were compared statistically. Results are shown in Supplemental Table 1.

There was a significant effect of genotype on global fluxes across skeletal muscle proteins in sedentary animals (Suppl. Fig1A, DESsed to WTsed: 29/42 with higher FSR in DESsed, $p<0.01$), but this effect was lost when comparing genotype across exercised groups (Suppl. Fig1B; DESrun to WTrun: 25/42 with higher FSR in DESrun, NS). When comparing the effect of exercise on the global skeletal muscle proteome, 74% of proteins showed higher FSR values (31/42, $p<0.001$) in the WTrun group when compared to WTsed (Suppl. Fig1C). In the Desmin R349P mutants, 71% (30/42, $p<0.005$) of proteins had higher FSR value in DESrun when compared to DESsed (Suppl. Fig1D). Further analysis compared the quantitative effects of exercise in DES vs WT groups. Of the 42 proteins analyzed, 31 had a response to exercise where FSR values changed in the same direction in both DES and WT groups ($p<0.001$, Suppl. Fig2) and could therefore be compared for quantitative magnitude of change. Of the 31 proteins that changed in the same direction in WT and DES groups, only 11 proteins changed in a quantitatively greater way in the DES group than in the WT group (Suppl. Fig2, NS). Overall, these results indicate that the effect of desmin mutation on FSR values across the skeletal muscle proteome in sedentary animals is modest yet significant, but that the differences in response to an endurance-based exercise routine does not significantly enhance the effect in the DES mutants when compared to their sedentary counterparts.

After correction for multiple comparisons, a small number of individual proteins showed statistically significant differences between groups. For the WTrun compared to the WTsed group, these were: *Beta-Enolase, Gamma-Enolase, Lactate Dehydrogenase alpha.*

For the DESrun compared to the DESsed group, these were: *Beta-Enolase, Phosphoglucomutase-1, ATP Synthase subunit Beta, Superoxide Dismutase [Cu-Zn].*

Methods

Deuterium oxide labelling

Animals were stable isotope labeled with deuterium oxide using previously published protocols with modifications [1, 2]. Briefly, endogenous deuterium levels were raised to about 4 % by an intraperitoneal injection of 0.014 mL $*g^{-1}$ bodyweight deuterium oxide (99.8 % + Atom D, Euriso-Top GmbH Saarbrücken) and 0.9 % NaCl. Deuterium levels were maintained via drinking water (4 % deuterium oxide). The stable isotope labeling started five days before collection and an independent group of unlabeled rats served as a control.

Body water enrichment analysis

Rat serum and liver were distilled overnight upside down on a bead bath at 85°C to evaporate out body water. Deuterium present in the body water were exchanged onto acetone, and deuterium enrichment in the body water was measured via gas chromatography mass spectrometry (GC-MS) [6].

LC-MS analysis

Trypsin-digested peptides were analyzed as described previously [4]. Proteins were clustered with the Functional Annotation Tool of the NIAID/NIH Database for Annotation, Visualization and Integrated Discovery (DAVID) version 6.8 [5]. For proteome-wide analysis, the binomial test and individual protein comparisons across groups with Benjamini-Hochberg correction for multiple comparisons were used. Proteins were included in the statistical analyses that met pre-established analytic criteria [3, 4] and for which at least 2 animals in each group had data.

Summary

42 proteins met rigorous data filtering criteria and were common among all 4 groups. Statistical analysis showed a significant effect of genotype on skeletal muscle protein flux in sedentary conditions. However, these differences were lost after downhill running. Exercise increased protein flux in both WT and DES animals. While key proteins of glycolysis, mitochondria and thick filaments increased their synthetic rates with running in WT, these proteins were higher at baseline and did not change with running in DES.

References

1. Busch R, Kim YK, Neese RA, Schade-Serin V, Collins M, Awada M, Gardner JL, Beysen C, Marino ME, Misell LM, Hellerstein MK (2006) Measurement of protein turnover rates by heavy water labeling of nonessential amino acids. Biochim Biophys Acta - Gen Subj 1760:730–744. doi: 10.1016/j.bbagen.2005.12.023

2. Gasier HG, Riechman SE, Wiggs MP, Previs SF, Fluckey JD (2009) A comparison of 2H2O and phenylalanine flooding dose to investigate muscle protein synthesis with acute exercise in rats. Am J Physiol - Endocrinol Metab 297. doi: 10.1152/ajpendo.90872.2008

3. Hedges L V (1981) Distribution Theory for Glass's Estimator of Effect Size and Related Estimators. J Educ Stat 6:107–128. doi: 10.2307/1164588

4. Holmes WE, Angel TE, Li KW, Hellerstein MK (2015) Dynamic Proteomics: In Vivo Proteome-Wide Measurement of Protein Kinetics Using Metabolic Labeling. In: Methods in Enzymology. Academic Press Inc., pp 219–276

5. Huang DW, Sherman BT, Lempicki RA (2009) Systematic and integrative analysis of large gene lists using DAVID bioinformatics resources. Nat Protoc 4:44–57. doi: 10.1038/nprot.2008.211

6. Yang D, Diraison F, Beylot M, Brunengraber DZ, Samols MA, Anderson VE, Brunengraber H (1998) Assay of low deuterium enrichment of water by isotopic exchange with [U-13C3]acetone and gas chromatography-mass spectrometry. Anal Biochem 258:315–321. doi: 10.1006/abio.1998.2632

Figure Captions

Suppl. Fig. 1 Comparison of proteome kinetics in skeletal muscle between DES and WT (in sedentary and exercised animals) Skeletal muscle (gastrocnemius) was collected and analyzed for global kinetic proteome effects of running. The 42 proteins that were measured in all groups and met analytical criteria are shown. (A) Comparison of DESsed to WTsed (29/42 with higher FSR in DESsed, $p<0.01$); (B) Comparison of DESrun to WTrun (25/42 with higher FSR in DESrun, NS); (C) Comparison of WTrun to WTsed (31/42 with higher FSR in WTrun, $p<0.001$); (D) Comparison of DESrun to DESsed (30/42 with higher FSR in DESrun, $p<0.005$).

Suppl. Fig. 2 Differences in protein FSR values in response to exercise were not significantly greater in DES than WT Proteins that met analytical criteria in WT and DES were further assessed for quantitative response to exercise. Proteins whose FSR values changed in the same direction in response to exercise in both WT and DES groups were included (31/42, changed in the same direction, $p<0.001$). Proteins shown as having change values great than 0 had quantitatively greater change in the DES group while proteins with values less than 0 had greater change in the WT group. Of the 31 proteins that changed in the same direction, 11 proteins had a quantitatively greater change in FSR values in response to exercise in the DES group compared to WT group (NS).

Supplementary Materials. **Table 1. FSR values for all 42 proteins. * $p<0.05$ from WTsed, ƚ $p<0.05$ from DESsed**

Suppl. Figure 1

A. (DESsed to WTsed)

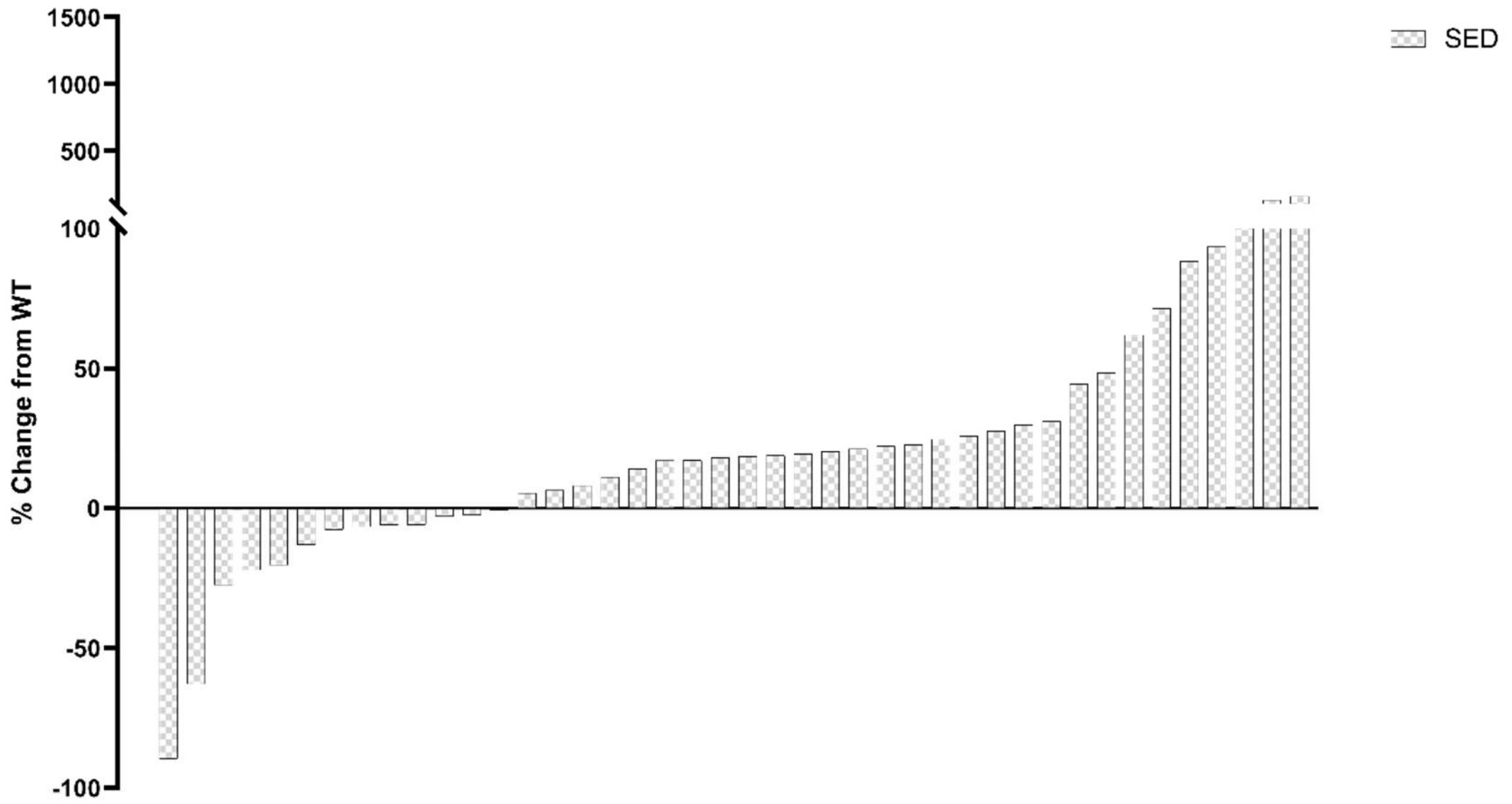

B. (DESrun to WTrun)

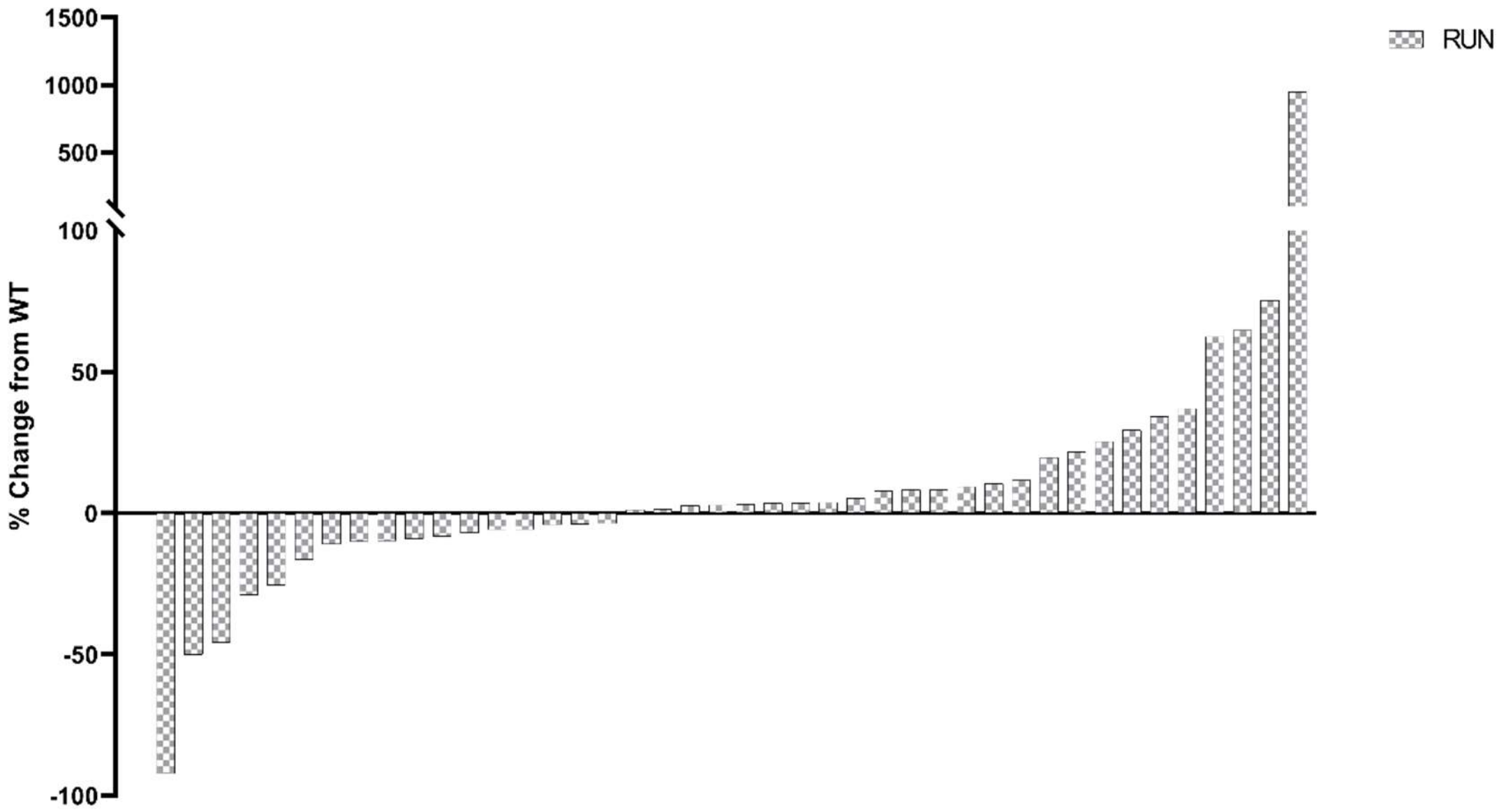

C. (WTrun to WTsed)

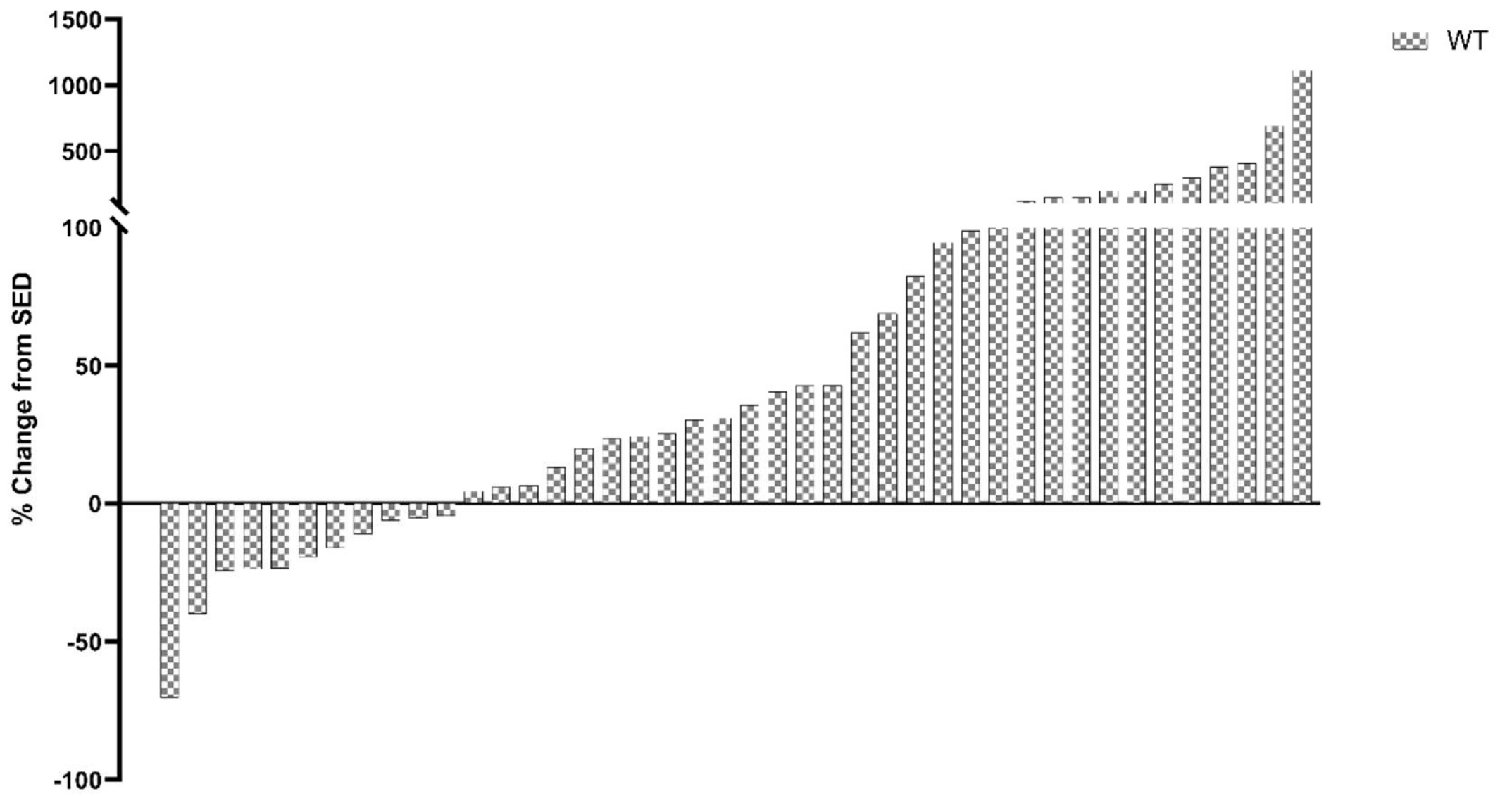

D. (DESrun to DESsed)

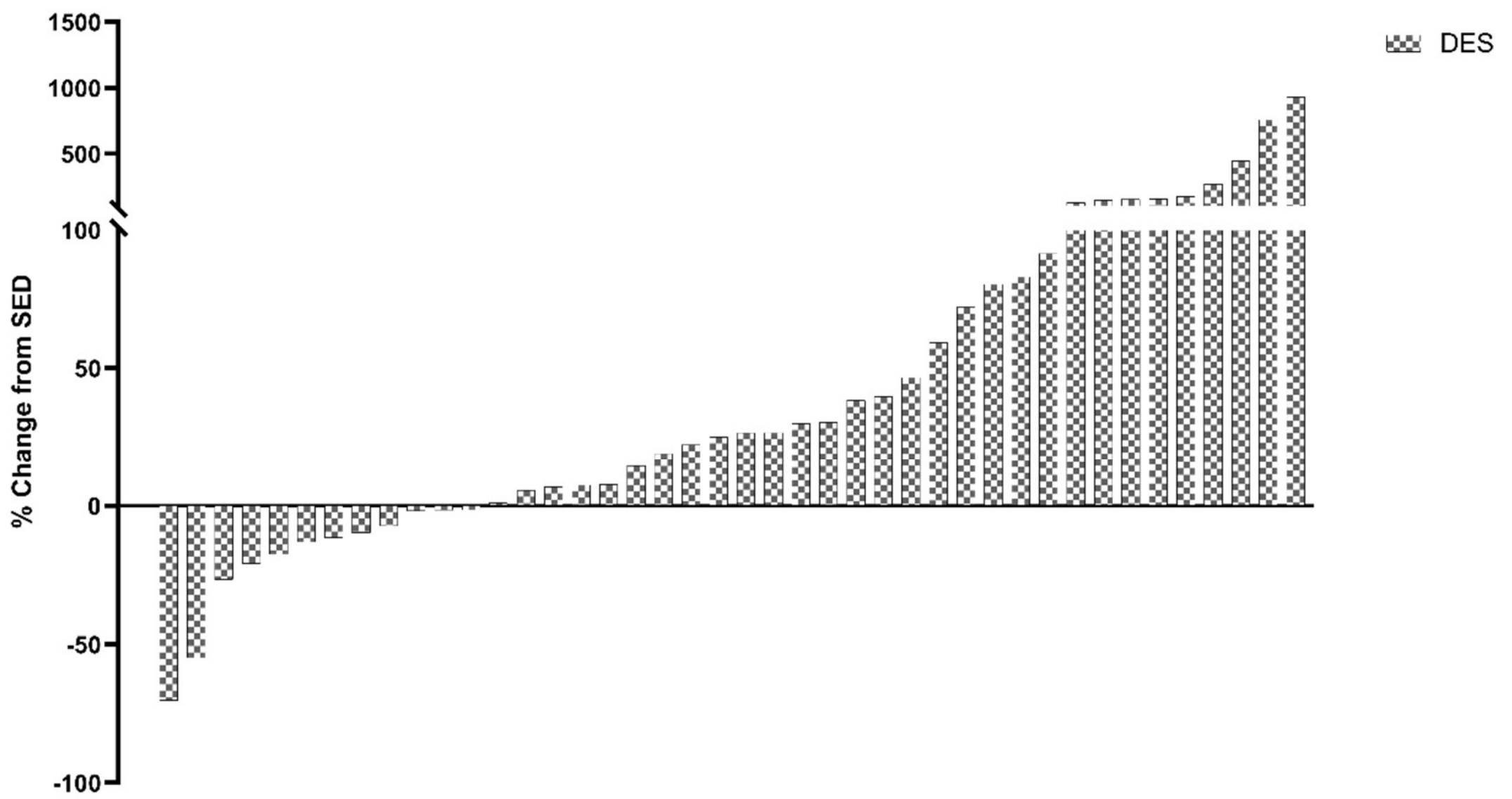

Suppl. Figure 2

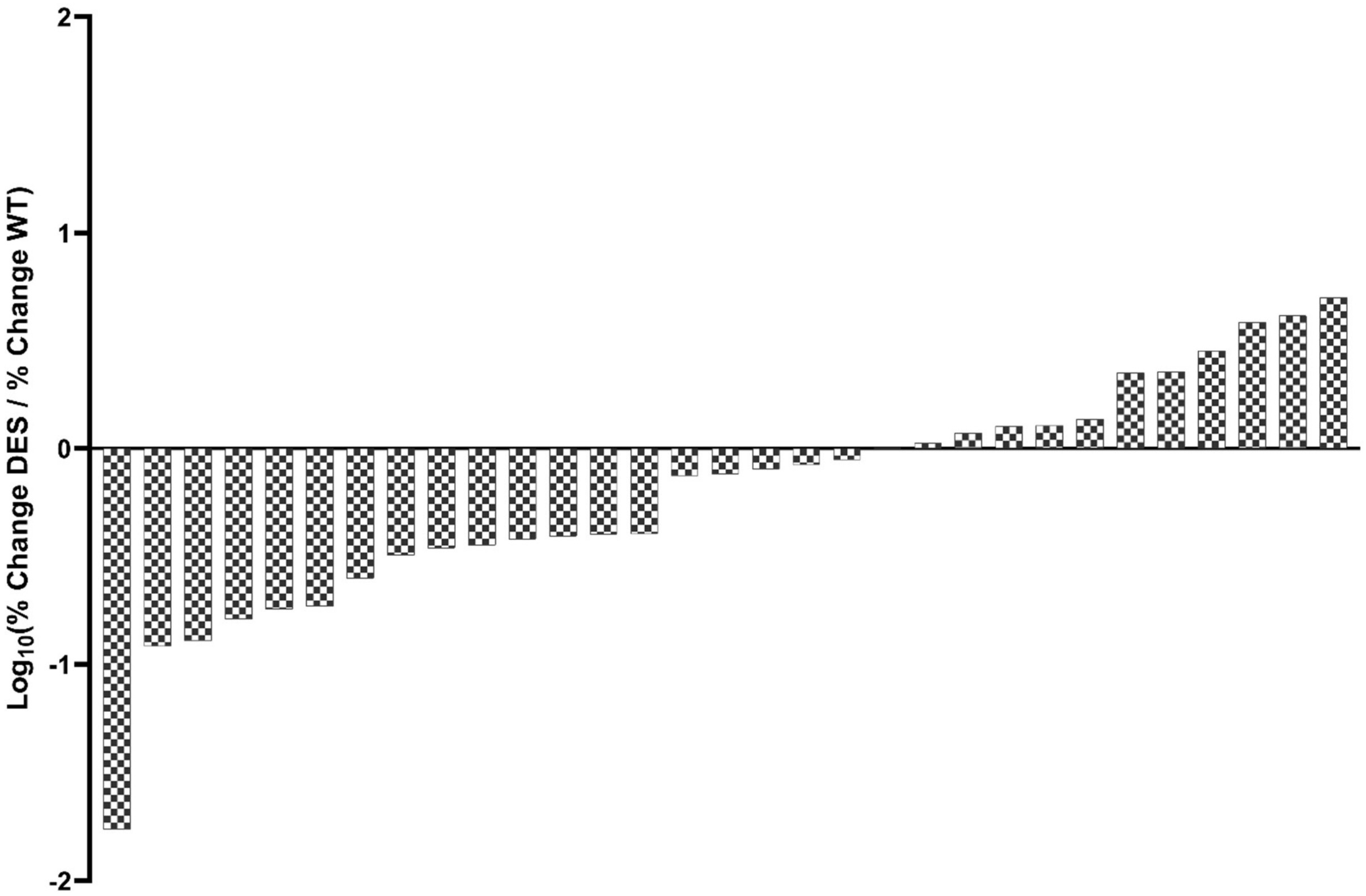

Table 1. FSR values for all 42 proteins. * p<0.05 from WTsed, ǂ p<0.05 from DESsed

			FSR values (turnover/day)			
			Wildtype		R349P Mutant	
Protein	Accession#	Mean Peptide Count	WT Sed	WT Run	DES Sed	DES Run
Beta-enolase	P15429	43.8	0.01097	*0.02180	0.01425	ǂ0.0261
Fructose-bisphosphate aldolase A	P05065	41.9	0.02294	0.03226	0.02705	0.03098
Creatine kinase M-type	P00564	36.4	0.01204	0.01579	0.01463	0.02044
Hemoglobin subunit alpha-1/2	P01946	29.0	0.01296	0.01602	0.01702	0.01506
Parvalbumin alpha	P02625	27.4	0.01220	0.01163	0.01390	0.01208
Albumin	P02770	24.1	0.19640	0.24645	0.19485	0.25333
Glyceraldehyde-3-phosphate dehydrogenase	P04797	23.6	0.01367	0.02309	0.01745	0.01719
Myoglobin	Q9QZ76	20.6	0.01531	0.01630	0.01614	0.02044
Phosphoglycerate mutase 2	P16290	18.6	0.01090	0.01139	0.01276	0.01560
Hemoglobin subunit beta-1	P02091	13.1	0.02785	0.02335	0.02214	0.02171
Phosphoglycerate kinase 1	P16617	12.8	0.00964	0.11708	0.01160	0.06335
Tropomyosin alpha-1 chain	P04692	11.1	0.02853	0.02678	0.02221	0.02898
Malate dehydrogenase, mitochondrial	P04636	10.3	0.02197	0.01951	0.02701	0.03215
Gamma-enolase	P07323	9.9	0.00886	*0.02193	0.01521	0.02424
Carbonic anhydrase 3	P14141	9.8	0.01726	0.02069	0.01592	0.01993
Hemoglobin subunit beta-2	P11517	9.0	0.03595	0.02715	0.02605	0.02753
Triosephosphate isomerase	P48500	8.2	0.00952	0.01541	0.01543	0.01560
Aconitate hydratase, mitochondrial	Q9ER34	6.8	0.02499	0.03396	0.03061	0.05521
L-lactate dehydrogenase A chain	P04642	6.4	0.00921	*0.03243	0.02419	0.03549
Alpha-enolase	P04764	6.0	0.01043	0.02031	0.01222	0.02104
Glucose-6-phosphate isomerase	Q6P6V0	5.6	0.01874	0.14877	0.01994	0.07420
Tropomyosin beta chain	P58775	5.6	0.03793	0.02888	0.01411	0.03516
Myosin-6	P02563	5.5	0.02604	0.03718	0.03095	0.03310
Myosin-7	P02564	4.9	0.02550	0.03645	0.03042	0.03277
Heat shock protein beta-1	P42930	4.7	0.05954	0.01774	0.05594	0.01668
Thymosin beta-4	P62329	4.3	0.14425	0.16305	0.17082	0.16796
Myosin light chain 1/3, skeletal muscle isoform	P02600	4.0	0.00666	0.03190	0.01255	0.02898
Nucleoside diphosphate kinase B	P19804	3.7	0.01741	0.05212	0.01699	0.04698
Myosin light chain 3	P16409	3.5	0.01748	0.03552	0.04085	0.03681
Alpha-crystallin B chain	P23928	3.4	0.08824	0.08346	0.11108	0.08796
ES1 protein homolog, mitochondrial	P56571	3.4	0.01830	0.01400	0.01707	0.14654
Malate dehydrogenase, cytoplasmic	O88989	3.3	0.01774	0.03238	0.03434	0.04346
Myosin-4	Q29RW1	2.9	0.02751	0.03422	0.03976	0.03277
Creatine kinase B-type	P07335	2.7	0.00982	0.03914	0.02009	0.02781
Troponin I, fast skeletal muscle	P27768	2.7	0.06530	0.05265	0.06136	0.05683
Phosphoglucomutase-1	P38652	2.4	0.02200	0.11225	0.01910	ǂ0.19687
Dihydrolipoyl dehydrogenase, mitochondrial	Q6P6R2	2.2	0.03968	0.05171	0.04408	0.04741
Musculoskeletal embryonic nuclear protein 1	Q80XX4	2.2	0.09121	0.09655	0.13550	0.09948
PDZ and LIM domain protein 3	Q66HS7	2.2	0.03144	0.09416	0.03925	0.10193
ATP synthase subunit beta, mitochondrial	P10719	2.1	0.05049	0.12462	0.05452	ǂ0.1392
60 kDa heat shock protein, mitochondrial	P63039	2.0	0.30759	0.18433	0.03201	0.01442
Superoxide dismutase [Cu-Zn]	P07632	2.0	0.01394	0.03100	0.01352	ǂ0.02591
